# A systematic review and synthesis of outcome domains for use within forensic services for people with intellectual disabilities^†^

**DOI:** 10.1192/bjpo.bp.116.003616

**Published:** 2017-02-13

**Authors:** Catrin Morrissey, Peter E. Langdon, Nicole Geach, Verity Chester, Michael Ferriter, William R. Lindsay, Jane McCarthy, John Devapriam, Dawn-Marie Walker, Conor Duggan, Regi Alexander

**Affiliations:** **Catrin Morrissey**, PhD, Division of Psychiatry and Applied Psychology, School of Medicine, University of Nottingham, Nottingham, and Complex and Forensic Service, Lincolnshire Partnership NHS Foundation Trust, Lincoln, UK; **Peter E. Langdon**, PhD, Tizard Centre, University of Kent, Canterbury, and Broadland Clinic, Hertfordshire Partnership University NHS Foundation Trust in Norfolk, Norwich, UK; **Nicole Geach**, MRes, Division of Psychiatry and Applied Psychology, School of Medicine, University of Nottingham, Nottingham, UK; **Verity Chester**, MSc, Department of Psychiatry, Partnerships in Care, Norfolk, and Norwich Medical School, University of East Anglia, Norwich, UK; **Michael Ferriter**, PhD, [Retired from] Forensic Division, Nottinghamshire Healthcare NHS Trust, Nottingham, UK; **William R. Lindsay**, PhD, Department of Psychology, University of the West of Scotland, UK, and Department of Psychology, The Danshell Group, UK; **Jane McCarthy**, FRCPsych, Department of Forensic and Neurodevelopmental Sciences (FANS), Institute of Psychiatry, Psychology & Neuroscience, King’s College London, London, UK; **John Devapriam**, FRCPsych, Department of Psychiatry, Leicestershire Partnership NHS Trust, Leicester, and Care Quality Commission, London, UK; **Dawn-Marie Walker**, PhD, Health Sciences, University of Southampton, Southampton, UK; **Conor Duggan**, OBE, FRCPsych, Institute of Mental Health, University of Nottingham, Nottingham, UK; **Regi Alexander**, FRCPsych, Department of Psychiatry, Partnerships in Care, Department of Psychiatry, Leicestershire Partnership NHS Trust, and Department of Psychiatry, University of Leicester, Leicester, UK

## Abstract

**Background:**

There is limited empirical information on service-level outcome domains and indicators for the large number of people with intellectual disabilities being treated in forensic psychiatric hospitals.

**Aims:**

This study identified and developed the domains that should be used to measure treatment outcomes for this population.

**Method:**

A systematic review of the literature highlighted 60 studies which met eligibility criteria; they were synthesised using content analysis. The findings were refined within a consultation and consensus exercises with carers, patients and experts.

**Results:**

The final framework encompassed three *a priori* superordinate domains: (a) effectiveness, (b) patient safety and (c) patient and carer experience. Within each of these, further sub-domains emerged from our systematic review and consultation exercises. These included severity of clinical symptoms, offending behaviours, reactive and restrictive interventions, quality of life and patient satisfaction.

**Conclusions:**

To index recovery, services need to measure treatment outcomes using this framework.

**Declaration of interest:**

None.

**Copyright and usage:**

© The Royal College of Psychiatrists 2017. This is an open access article distributed under the terms of the Creative Commons Attribution (CC BY) licence.

Following de-institutionalisation, most people with intellectual disabilities live fairly independent lives in the community. There are 900 000 adults with intellectual disabilities in England, and estimates suggest that only around 3035 (0.3%) receive treatment in psychiatric hospital settings, with about half of them being in forensic hospitals.^1^^–^^3^ The health expenditure in this sector belies the low numbers, and it is estimated at over 300 million pounds sterling per annum.^4^^,^^5^ However, there is limited empirical information on service-level outcome domains and indicators, which in turn limits the ability to measure the effectiveness of these services. This is of concern in a health climate focused on outcomes^6^ and ‘payment by results’, but is even more relevant because of the recent government initiative to fundamentally transform care for people with intellectual disabilities.^7^ Although Fitzpatrick *et al*^8^ have conducted a systematic review of outcome measures used in generic forensic mental health services, and Gilbody *et al*^9^ completed a similar review of outcome studies in mental health, there has been no such work for forensic services providing care to people with intellectual disabilities. Further, although there has been a marked focus on recovery from mental health services, there has been comparatively little focus on this construct and its measurement within psychiatric hospital settings for people with intellectual disabilities, including forensic services. Recovery is often construed as ‘getting better’ or ‘reducing symptoms’, and within the context of in-patient services for people with intellectual disabilities, where there is often a focus on person-centred support and normalisation, including living as independently as possible within the community, the concept remains unclear, but the issues are not dissimilar from the ‘recovery’ debates within wider mental health services. However, recovery in the context of forensic services for people with intellectual disabilities, while subjective, should nevertheless incorporate the connectedness; hope and optimism about the future; identity; meaning in life; and empowerment (CHIME) framework,^10^ bearing in mind that some associated factors may be more proximal for this population (e.g. offending behaviours and stigma associated with disabilities).

In order to address these shortcomings, this study had the single aim of identifying the domains that should be used to measure outcome from forensic services for people with intellectual disabilities. Within the context of this project, outcome was defined as occurring at the level of the service as a whole, rather than individual outcomes associated with a specific treatment or intervention. In other words, we were primarily interested in outcomes that could index change over time across the entire range of interventions offered by a service, rather than outcome from a specific intervention (e.g. medication or psychological treatment), as this represents the real world of service delivery. Our aim was achieved within the context of two interrelated and iterative work streams: (a) undertaking a systematic review of studies that focused directly or indirectly on measuring outcomes from forensic services for people with intellectual disabilities and synthesising the findings into an initial framework of outcome domains, and (b) taking our initial framework and refining further within the context of a consultation exercise with patients and carers, as well as a two-round Delphi exercise with experts.

## Method

### Systematic review

An initial outcome framework was developed following a systematic review of the literature that focused on outcomes from forensic services for people with intellectual disabilities. As a starting point, and following discussion within the research team, we initially envisaged outcomes as falling into one of the three areas that were defined by the Department of Health^11^ as representative of quality. They are as follows: (a) effectiveness (e.g. the impact of generic treatment on health), (b) patient safety (e.g. untoward events as a result of treatment) and (c) patient experience of care (e.g. satisfaction).

#### Search strategy

The search strategy aimed to identify studies from a range of sources. Electronic databases searched on 1 June 2015 included Medline, Psyc (INFO), Embase, AMED, HMIC, BNI and CINAHL. Search terms employed were based on those used for a previous Cochrane reviews, for intellectual disability^12^ and forensic/offenders.^13^ The full search terms, including ‘explode’ terms, keywords and text words are included within our supplementary material. The systematic review is registered in advance with PROSPERO (registration number: CRD42015016941).

In order to ensure that no relevant publications were missed, the grey literature (opengrey.eu) was also searched using the keywords. The ancestry method was used to find suitable studies within the references of eligible papers. The ancestry method means searching the reference lists of papers that met our eligibility criteria for any further papers that may not have been previously included. In addition, expert members of the project team were consulted in order to identify any key references not retrieved by the search strategy as well as in press or unpublished articles.

#### Study selection and eligibility criteria

Duplicate studies were removed, and titles and abstracts of articles were screened against the eligibility criteria independently by two members of the research team (C.M. and N.G.). Any disagreements were resolved by a third reviewer (M.F.). Studies were included that (a) were published after 1980, as our initial searches revealed there was little relevant literature available before 1980; we opted to use this cut-off date to reduce the number of returned ineligible papers; (b) were in any language, as translations were obtained; (c) made use of any type of quantitative method; (d) involved adults within intellectual or autism spectrum disorders; (e) who were older than 18 years of age; and (e) had current or past use of forensic services for people with intellectual disabilities, including community-based forensic services. Forensic services were defined according to the bed categories defined by the Royal College of Psychiatrists.^3^ This means that we included papers where the participants were either living within a high, medium or low secure in-patient forensic health service, or a forensic rehabilitation service, or they were living in the community, but receiving a service from a community-based forensic service. Studies were excluded if they only evaluated the effects of a specific intervention or treatment programme (e.g. randomised control trial of a medication or psychological treatment group), rather than examining outcomes at a service level.

**Fig. 1:**
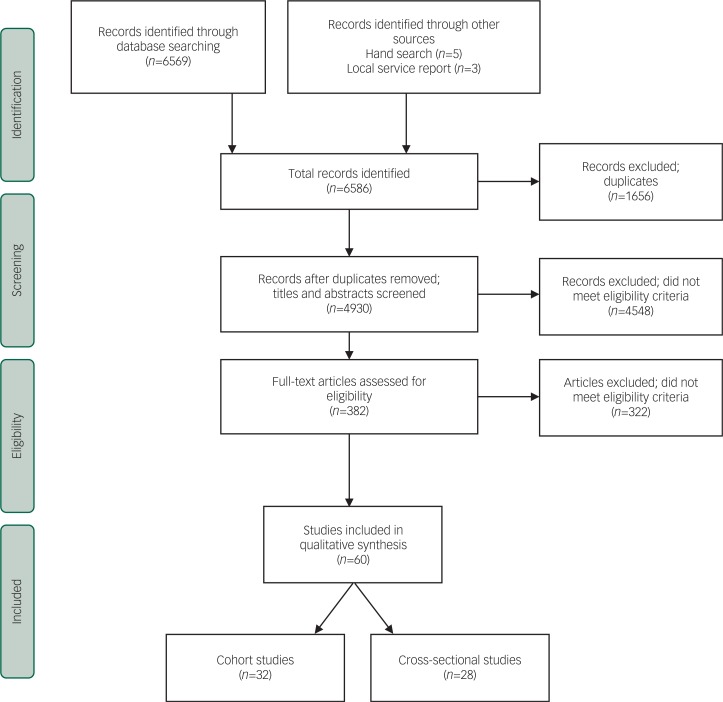
Flow chart of study selection process.

Sixty studies met the inclusion criteria. None of the included studies were randomised controlled trials or meta-analyses. Twenty-eight studies were cohort outcome studies with follow-up from 1 to 20 years, and a further 32 were cross-sectional studies which reported service-level outcome data at one point in time. Several of these studies made use of the same or overlapping samples of participants, but as we did not make use of meta-analytic methods, this did not erroneously affect precision. The large majority of the studies included were from the UK, with only two studies originating elsewhere. Most studies made use of samples of men, as only two studies included women. Figure 1 depicts a flowchart outlining the study selection process and the number of studies identified at each stage.

#### Data extraction and analysis

Using a structured form, data were extracted from the included articles. Specifically, details regarding the sample, design, service type, methods, outcome domains and specific measures were obtained and coded. Content analysis was used to synthesise the outcome domains with reference to the three areas of quality as defined by the Department of Health,^11^ namely: (a) effectiveness, (b) patient safety and (c) patient experience. These three areas were used as an *a priori* superordinate framework. A process of refining and grouping similar outcome sub-domains together was then undertaken by two researchers. This led to the construction of a ‘framework’ to describe the outcome domains extracted from the eligible studies. This process is best described as both directed and summative content analysis because the process started with an *a priori* theoretical stance pertaining to service quality, followed by both counting and coding the extracted data, which was then interpreted within the context of our *a priori* theoretical stance.^14^ This methodology was advantageous because it allowed us to identify key concepts, consider their context and underlying meaning within and across studies, and develop coding variables, which were then refined into sub-domains.

### Consultation exercise

#### Consultation groups

Following the completion of our systematic review, and the development of our initial outcome framework, we undertook three consultation groups with patients and one consultation group with carers to further consider and refine our outcome framework. Two of our patient groups took place within a high secure hospital in England, whereas the remaining groups took place within both a low secure hospital and a medium secure hospital, also in England. Participants were approached by the researchers, and the purpose of the group was explained using information sheets. Participants who agreed to take part provided informed consent. However, we were advised by our associated Research Governance office, within our National Health Service (NHS) Trust, that NHS Research Ethics opinion was not required for this project. Our groups included 3 women, 1 transgendered person and 11 men. For our consultation group involving carers, we recruited four participants from an existing carer group within a secure hospital, whereas two participants were recruited who were not part of this group. The carer participants had family members detained within three different secure hospitals.

##### Analysis

A semi-structured topic guide was used which was based around our initial outcome framework as a method to structure the conversations within our groups. Participants were encouraged to consider and discuss our initial framework, make modifications and choose outcomes they considered most important. The discussions were recorded and fully transcribed. The transcriptions were analysed using both directed and summative content analysis.^14^ As with our systematic review, this methodology allowed us to identify key concepts, context and meaning within transcripts, which were interpreted within our proposed framework. Any changes, or newly identified outcome sub-domains, were incorporated within our superordinate framework.

#### Delphi exercise

The Delphi method^15^^,^^16^ is an iterative and multi-staged structured process that can be used to develop group consensus. We made use of a two-round online Delphi exercise with expert clinicians, researchers and commissioners with experience of working within forensic services for people with intellectual disabilities. Information about the study was advertised within the communication networks of existing stakeholder organisations within the UK (e.g. British Psychological Society). All participants were provided with information to help them make a decision as to whether they wished to take part in the study. Participants were presented with the revised outcome framework developed following our patient and carer consultation exercise. They were then invited to rate the importance of each sub-domain within each of the three superordinate domains along a 5-point Likert scale, where 1 was ‘not important’ and 5 was ‘extremely important’. Participants were also asked for their expert opinion about each sub-domain and whether they thought any additional outcome measures needed to be added. Finally, participants were asked to indicate the five sub-domains they considered to be the most important measures of outcome.

Following the completion of the first round, participants were invited to consider the responses of the group and re-consider their previous ratings. Those sub-domains with a mean rating of four or more were taken through to the second round, and participants re-rated their importance along the same 5-point Likert scale. Participants were invited to select up to five sub-domains they perceived to be the most important. All participants were reminded that they did not have to change their original responses.

##### Participants

Seventeen participants took part in the first Delphi round, with 15 taking part in the second round. Nine participants were psychologists, seven were psychiatrists and one was a nurse. Participants were eligible to take part in the Delphi exercise if they were a clinician, researcher or commissioner with experience of working with forensic services for people with intellectual disabilities. Two participants identified themselves as having responsibility for commissioning, whereas a further two identified themselves as having both clinical and academic responsibilities.

## Results

### Systematic review

Using content analysis, data from eligible studies were extracted and categorised within the overarching superordinate domains: (a) effectiveness, (b) patient safety and (c) patient and carer experience. The complete list of identified sub-domains that emerged following our analysis, along with the associated studies, is found in Tables 1–6. For simplicity, studies have been divided into cohort, retrospective cohort, cross-sectional or case study designs. These findings were synthesised into our initial framework of outcomes which was taken forward and used within our consensus exercises (Table 7).

**Table 1:** Summary of studies presenting data on the outcome domain of effectiveness (cohort studies)

Study and setting	Design	*N*	Outcome sub-domain	Measure/indicator
Alexander *et al*^17^ Medium secure	Retrospective cohort study Follow-up: up to 13 years	64	Treatment response/outcome Discharge pathway Readmissions Relapse in MH Reoffending ‘Offending-like’ behaviour	Level of CPA support, MHA status and CGIS Level security/type of placement at discharge % of patients readmitted to the same unit % of patients who experienced a relapse in MH symptoms Number of patients formally reconvicted, cautioned or had police contact Behaviour which could be classed as an offence but did not lead to police contact
Alexander *et al*^a^^,^^18^ Medium secure	Retrospective cohort study Discharged patients Follow-up: 6 years	138	Length of stay Discharge outcome Discharge pathway	Mean and median number of days % of positive (move to a lower level of security) or negative (move to higher level of security) discharges Level security/type of placement at discharge
Alexander *et al*^5^ Medium secure	Retrospective cohort study Follow-up: 6 years	138	Length of stay Discharge outcome	Mean and median number of days % discharged successfully (to a lower level of security) or not (to same/higher level of security)
Alexander *et al*^19^ Medium secure	Retrospective cohort study (arson *v.* non-arson) Follow-up: 6 years	30	Length of stay Discharge pathway	Discharged patients only; mean and median days % of discharged patients who moved to either a lower or same/higher level of security
Alexander *et al*^20^ Medium secure	Retrospective cohort study (Discharged patients. PD *v.* ID *v.* PD+ID) Follow-up: up to 9 years	145	Length of stay Clinical symptoms Risk assessment Reoffending Time to reoffend	Median number of days and age on discharge PCL:SV pre-treatment scores (clinician rated) HCR-20 pre-treatment scores (clinician rated) Reconvictions at 1, 2 and 5 years follow-up. Categorised as serious/violent offending (as defined by Home Office) The difference between discharge date and reconviction date for subsequent offence (median)
Ayres & Roy^21^ Community	Case series (70% male) Follow-up: up to 3 years	26	Cost Level of support	Average saving cost per patient (based on hourly rate of support) and for the service over 3 years Hours per annum providing both direct/indirect support
Barron *et al*^22^ Community	Cohort study Follow-up: 9.5 months average	61	Clinical symptoms Treatment engagement Reoffending ‘Offending-like’ behaviour	ABC and Mini PAS-ADD (clinician rated) Categorised treatment use and% with history of service use Number of patients who reoffended; mean number of offences per patient Telephone call with carers regarding police contact
Benton & Roy^23^ Community	Cohort study Follow-up: up to 3 years	113	Reoffending Discharge pathway Discharge outcome Prevention of in-patient admissions	Number of patients arrested, reconvicted and cases dropped Level security/type of placement at discharge % of referrals discharged Number of patients whose in-patient admission was prevented via community treatment
Butwell *et al*^24^ High secure	Cohort study Follow-up: 10 years	Up to 278	Length of stay Discharge pathway	Median and mean years, calculated per episode Level security/type of placement at discharge
Day^25^ Medium secure	Cohort study Follow-up: 3 years	20	Length of stay Treatment response Discharge pathway Discharge outcome Readmission Reoffending	Mean number of months Categorised as either good (settled and co-operative), fair (continuing lesser problems) or poor (severe problems) % of patients discharged to rehab villa, community or hostel Level of adjustment at follow-up, based on personal knowledge, hospital notes and liaison with involved agencies % of patients readmitted to the same unit Number who reoffended or returned to prison
Dickens *et al*^26^ Medium and low secure	Retrospective cohort study Follow-up: 15 months	48	Length of stay Clinical symptoms	Mean number of days HoNOS-Secure: change between baseline and final rating. Quartile points used to allow for different lengths of stay (clinician rated)
Fitzgerald *et al*^b^^,^^27^ Medium secure	Retrospective cohort study Follow-up: 2 years	145	Reoffending Risk assessment	Home Office records;% of patients with both general and violent offences. OGRS
Gray *et al*^28^ Medium secure	Retrospective cohort study Follow-up: 2 years	115	Reoffending Risk assessment	Home Office records:% of patients with both general and violent offences HCR-20,VRAG, PCL: SV (rated once)
Halstead *et al*^29^ Medium secure	Cohort study Discharged patients Follow-up: up to 13 years	35	Length of stay Treatment outcome Discharge outcome Discharge pathway Relapse in MH Readmissions Offender-like behaviour Reoffending	Mean and median months Rated as either good (risks reduced, safe for discharge), some (progress made in some areas, risk remains), none (no change) or poor (worse than admission) % of patients discharged Level security/type of placement at discharge and follow-up Recurrence of symptoms of illness or challenging behaviour % of patients re-admitted to hospital Behaviour which could be interpreted as an offence Number of patients reconvicted
Lindsay *et al*^c^^,^^30^ Community	Cohort study Follow-up: 13 years	62	‘Offending-like’ behaviour Reoffending	Percentage of patients ‘suspected of’ reoffending Percentage of patients with clear evidence of reoffending
Lindsay *et al*^31^ Community	Cohort study Follow-up: 7 years	184	Reoffending Harm reduction	Number of patients with clear evidence of reoffending Decrease in the number of incidents 2 years before referral *v.* incident data at follow-up
Lindsay *et al*^44^ Community Females only	Case series Follow-up: 3 years	18	Reoffending	Number of patients with clear evidence of reoffending
Lindsay *et al*^32^ Community	Cohort study Follow-up: 12 years	247	Reoffending Harm reduction	Number of patients with clear evidence of reoffending Decrease in the number of incidents 2 years before referral *v.* incident data at follow-up
Lindsay *et al*^33^ Community	Cohort study Follow-up: up to 20 years	309	Reoffending Harm reduction	Number of patients with clear evidence of reoffending Standardised service incident report data; number of offences committed in the 2 years before referral *v.* follow-up reoffending data
Lindsay *et al*^34^ Mixed services	Cohort study Follow-up: 2 years	197	Discharge pathway	Number of patients discharged and level security/type of placement at discharge, 1-year and 2-year follow-up
Linhorst *et al*^35^ USA Community service	Cohort study Follow-up: 6 months	252	Reoffending Treatment response/engagement	Local law agency data and arrests frequencies used Number of patients who completed treatment
Marks^36^ [unpublished thesis] Medium secure	Retrospective cohort study Follow-up: 4 years	28	Reoffending Length of stay Discharge pathway	% of patients who reoffended (even if this did not lead to police contact) determined by interview with current care team Mean and median number of years % of patients in high, medium, low and community services
Morrissey & Taylor^37^ High secure	Case reports Follow-up: 2 years	13	Clinical symptoms Discharge outcome	YSQ (patient rated), IPDE and PCL:SV (clinician rated): change in mean scores Number of patients remaining in treatment, discharged to medium secure or transferred to another high secure ward
Morrissey *et al*^38^ High secure	Retrospective cohort study (ID *v.* other non-ID services) Follow-up: 5 years	70	Risk assessment	HCR-20: change in mean scores
Morrissey *et al*^39^ High secure	Prospective cohort study Follow-up: 2 years	73	Discharge outcome Risk assessment Clinical symptoms	% of patients who made positive (move to a lower level of security) or negative (the same level of security) progress HCR-20 PCL-R (clinician rated)
Morrissey *et al*^40^ High secure	Retrospective cohort study (Two cohorts: in-treatment and admission cohort) Follow-up: up to 6 years	68 and 24	Length of stay Incidents Clinical symptoms Discharge pathway Discharge outcome Readmission	Median number of years Frequency per year in the first 4 years of treatment using hospital incident records. Mean number of violent incidents per patient/year EPS-BRS (clinician rated) & EPS-SRS (patient rated) Level security/type of placement at discharge % of patients who moved to a lower level of security Number of returns from trial leave
Palucka *et al*^41^ Canadian in-patient unit	Retrospective cohort study Follow-up: up to 9 years	20	Length of stay Discharge outcome Discharge pathway Reoffending	Median number of days Number of patients discharged Level security/type of placement at discharge Any criminal justice involvement (even if charges were not pressed)
Reed *et al*^d^^,^^42^ Low secure	Retrospective cohort study Follow-up: up to 14 years	45	Length of stay Discharge pathway Discharge outcome Incidents	Mean number of weeks Level security/type of placement at discharge Positive (discharged to a level of lower security) or negative (discharged to a level of higher security) outcome Collected from hospital incident records. Incidents at baseline (week 6 to 10 of stay) were compared to end of stay (last 4 weeks of treatment). Frequency (total number of incidents per month) was adjusted for length of stay. Change in incidents calculated per person per week
Xenitidis *et al*^43^ Low secure	Retrospective cohort study Follow-up: up to 11 years	64	Length of stay Discharge pathway Discharge outcome Incidents	Mean number of months Level security/type of placement at discharge Good (discharged to community) or bad (not placed in a community setting) outcome Collected from hospital incident records. Incidents at baseline (week 6 to 10 of stay) were compared to end of stay (last 4 weeks of treatment). Frequency (total number of incidents per month) was adjusted for length of stay.

ABC, Aberrant Behaviour Checklist; Mini PAS-ADD, Mini Psychiatric Assessment Schedules for Adults with Developmental Disabilities; YSQ, Young Schema Questionnaire; IPDE, International Personality Disorder Examination; PCL-R, Psychopathy Checklist – Revised; EPS-BRS. Emotional Problems Scale – behaviour rating scale; EPS-SRS, Emotional Problems Scale – self report scale; CPA, Care Programme Approach; MHA, Mental Health Act; MH, mental health; CGIS, Clinical Global Impressions Scale; PCL:SV, Psychopathy Checklist: Screening Version; HCR-20, Historical, Clinical, Risk Management-20; HoNOS, Health of the Nation Outcome Scale; OGRS, Offender Group Reconviction Scale; VRAG, Violence Risk Appraisal Guide.

a The sample in Alexander *et al*^18^ overlaps with that of Alexander *et al*,^5^^,^^19^ and Esan *et al*.^4^

b The sample in Fitzgerald *et al*^27^ overlaps with that of Gray *et al*^28^

c The sample in Lindsay *et al*^30^ overlaps with that of Lindsay *et al*.^31^^–^^33^^,^^44^^,^^45^

d The sample in Reed *et al*^42^ overlaps with that of Xenitidis *et al*.^43^

**Table 2:** Summary of studies presenting data on the outcome domain of effectiveness (cross-sectional studies)

Study and setting	Design	*N*	Outcome sub-domain	Measure/indicator
Ajmal^46^ High secure	Cross-sectional	79	Clinical symptoms	GSI and RSES (patient rated)
Beer *et al*^47^ Low secure	Cross-sectional	59	Placement appropriateness Clinical symptoms	Percentage of patients assessed as requiring a less secure placement SBS (clinician rated)
Beer *et al*^48^ Low secure	Cross-sectional	68	Length of stay Placement appropriateness Clinical symptoms	Mean number of months Percentage of patients requiring less secure care. Main reason for delayed discharge via the Royal College of Psychiatrists Research & Development questionnaire HoNOS-Secure (clinician rated)
Chaplin *et al*^49^ Low secure	Cross-sectional	22	Risk assessment Incidents Length of stay	HCR-20 median scores Average per patient at 3 monthly intervals. Coded for severity using theMOAS. Median number of incidents Median number of days
Chilvers & Thomas^50^ Medium secure	Cross-sectional (M *v.* F)	77	Clinical symptoms	NAS-PI scores (patient rated)
Crossland *et al*^51^ High, medium and low secure	Cross-sectional	60	Length of stay	Median number of months
Dickens *et al*^52^ Medium and low secure	Cross-sectional 16-month period	68	Incidents	Severity rated by the individual completing the form as either: near miss, minor, moderate, high or very high Incidents/total bed days × 100. Average number of incidents per 100 occupiedbed days, time of incident, number of violent/aggressive incidents and total number of incidents
Esan *et al*^4^ Medium and low secure	Cross-sectional (ASD *v.* non-ASD)	114	Length of stay Discharge outcome Level of supervision/discharge pathway	Mean and median months for both discharged and in-treatment patients Number of patients with a good (move to a lower level of security) or poor (move to a higher level of security) outcome Number of patients who were informal,under a MHA section, guardianship or supervised discharge
Fitzgerald *et al*^53^ Medium and low secure	Cross-sectional	136	Incidents Risk assessment	Number of patients involved inincident in 6-month period VRAG and HCR-20
Hall *et al*^54^ Medium and low secure	Cross-sectional	136	Treatment needs Security need Delayed discharge Length of stay Incidents	Clinician ratings Reference group ratings of appropriate security level Number of patients no longer requiring current security level, main obstacle to progress Maximum and average years per level of security Number of patients involved in anincident in 6 months period
Hogue *et al*^55^ High, medium, low and community	Cross-sectional	228	Clinical symptoms	EPS-BRS (clinician rated)
Johnson^56^ Medium and low secure	Cross-sectional	44	Clinical symptoms Length of stay	RSES and EBS (patient rated) Mean number of months
Kellett *et al*^57^ High secure	Cross-sectional	45	Clinical symptoms	BSI (patient rated)
Lindsay *et al*^45^ Community	Cross-sectional	52	Offender-like behaviour Reoffending	Percentage of patients suspected of reoffending Percentage of patients with ‘clear evidence’ of reoffending
Lindsay *et al*^58^ High, medium and low secure	Cross-sectional	212	Risk assessment Clinical symptoms	HCR-20, VRAG, Static-99, SDRS, RM-2000 EPS-BRS (clinician rated)
Lindsay *et al*^59^ High, medium, low secure and community	Cross-sectional	197	Risk assessment	VRAG and Static-99
Lofthouse *et al*^60^ Rehabilitation, acute admission and residential home	Cross-sectional 5 months of data	64	Length of stay Risk assessment Incidents	Mean number of years CuRV Aggression defined as acts of physical violence,aggression, force to hurt or damage to staff,peers or environment. Included verbal abusewhich was aggressive, threatening or caused offence. Two researchers rated each incidentas: ‘aggression present’ or ‘aggression absent’. Number of patients who were aggressive in month 1 versus month 5
Mansell *et al*^61^ Medium and low secure	Cross-sectional NHS versus private provider units	1891	Delayed discharge Incidents	Percentage of patients who had completed treatment but did not have any plans to leave the service in the next month. Average frequency where a patient was hurt by a patient or staff member(per patient over a 6-month period)
McMillan *et al*^62^ Medium secure	Cross-sectional 6-month period	124	Risk assessment Incidents	MDT ratings per patient on risk of physical violence (scale of 0-8) and number of times patient had been violent in 6 months prior to risk assessment Author coded each description based on explicit criteria and guidelines. e.g. physical violence (attempted, contact between assailant or object and victim, evidence of physical harm to victim or attendance of medical personnel). Coded from computerised hospital database
Morrissey *et al*^39^ High secure	Cross-sectional 12-month period	60	Incidents Risk assessment Clinical symptoms	Coded as either interpersonal physical aggression or verbal aggression/aggression to property. Further rated as low, medium or high risk of harm. Number of patients involved in an aggressive incident HCR-20 PCL-R and EPS-BRS (clinician rated)
O’Shea *et al*^63^ Medium, low and rehabilitation	Cross-sectional	109	Risk assessment Incidents	HCR-20 Hospital records in 3-month period following risk assessment for aggression and self-harm.Coded using Overt Aggression Scale. Rated on severity (1-4). Number of patients involved in any incident
Perera *et al*^64^ Medium and low secure	Cross-sectional	388	Length of stay Delayed discharge	Median number of years and percentage of patients who had stayed longer than 5 years Percentage of patients assessed as requiring a less secure placement
Thomas *et al*^65^ High secure	Cross-sectional	102	Length of stay Delayed discharge Security need Treatment needs	Mean and median number of years Percentage of patients assessed as requiring a less secure placement and main reason for this SDTN scale completed by key worker and responsible clinician CANFOR-Short and CANDID-Short. Average number of needs and unmet needs
Uppal & McMurran^66^ High secure	Cross-sectional (ID sample included in wider data-set) 15-month period of incidents	396	Incidents	Hospital computerised reporting system. Coded as per Department of Health: Category A: major incidents (e.g. abscond, hostage taking); Category B: serious incidents (e.g. serious assault involving a weapon, attempted suicide); Category C: untoward incidents (e.g. attempted abscond, assault without a weapon); Category D: all other incidents (minor assault and verbal abuse) Most frequent location and time of incident Percentage of incidents which were violent and which were self-harm Average monthly figure generated

GSI, Global Severity Index; RSES, Rosenberg Self Esteem Scale; SBS, Social Behavioural Schedule; MOAS, Modified Overt Aggression Scale; NAS-PI, Novaco Anger Scale and Provocation Inventory; ASD, autistic spectrum disorder; VRAG, Violence Risk Appraisal Guide; RSES, Rosenberg Self-Esteem Scale; EBS, Evaluative Beliefs Scale; BSI, Brief Symptom Inventory; SDRS, Short Dynamic Risk Scale; RM-2000, Risk Matrix 2000; CuRV, Current Risk of Violence; MDT, Multidisciplinary team; SDTN, Security, Dependency and Treatment Needs Scale; CANFOR, Camberwell assessment of need – forensic version; CANDID, Camberwell Assessment of Need for Adults with Developmental and Intellectual Disabilities; ID, intellectual disability.

**Table 3:** Summary of studies presenting data on the outcome domain of patient safety (cohort studies)

Study and setting	Design	*N*	Outcome sub-domain	Measure/indicator
Alexander *et al*^19^ Medium and low secure	Retrospective cohort study Follow-up: 6 years	30	Seclusion, restraint and intensive observations	Mean number of episodes per patient/month
Alexander *et al*^18^ Medium secure	Retrospective cohort study Follow-up: 6 years	138	Seclusion, restraint and intensive observations	Mean number of episodes per patient/month (adjusted for length of stay)
Alexander *et al*^5^ Medium secure	Retrospective cohort study Follow-up: 4 years	138	Seclusion, restraint and intensive observations	Mean and median episodes per patient/month
Ayres & Roy^21^ Community	Case series Follow-up: up to 3 years	26	Medication	Case study: reduction in frequency of use of *pro re nata* medication
Butwell *et al*^24^ High secure	Cohort study Follow-up: 10 years	Up to 278	Death	Frequency of episodes (*n* and % of all patients)
Morrissey & Taylor^37^ High secure	Cohort study Follow-up: 2 years	13	Seclusion	Hours per patient for every 6 months of treatment
Reed *et al*^42^ Low secure	Retrospective cohort study Follow-up: up to 14 years	45	Seclusion, restraint and relocation	Episodes at baseline (weeks 6 to 10 of treatment) were compared to end of stay (last 4 weeks of treatment) Mean monthly rates calculated to control for length of stay
Xenitidis *et al*^43^ Low secure	Retrospective cohort study (4) Follow-up: up to 11 years	64	Seclusion	Episodes at baseline (week 6 to 10 of treatment) compared to end of stay (last 4 weeks of treatment)

**Table 4:** Summary of studies presenting data on the outcome domain of patient safety (cross-sectional studies)

Study and setting	Design	*N*	Outcome sub-domain	Measure/indicator
Esan *et al*^4^ Medium and low secure	Cross-sectional (ASD *v.* Non-ASD)	114	PRN usage Restraint, seclusion and intensive observations	Mean number of episodes (total frequency divided by total number of months of stay to provide an average monthly figure)
Mansell *et al*^61^ Medium and low secure	Cross-sectional 6-month period	1891	PRN usage Seclusion, restraint, locked areas Access to healthcare	Average number of episodes Average number of episodes % of units who reported a delay in patients accessing primary (nurse/dentist) healthcare
Mason^67^ High secure	Cross-sectional 12-month period	36	Seclusion	Number of patients secluded; average number of seclusion episodes per patient/year; reason for seclusion and distress-related behaviours after seclusion

ASD, autism spectrum disorder; PRN, *pro re nata*.

**Table 5:** Summary of studies presenting data on the outcome domain of patient and carer experience (cohort studies)

Study and setting	Design	*N*	Outcome sub-domain	Measure/indicator
Fish & Lobley^68^ Community	Cohort study (4) Follow-up: 1 year	20	Quality of life	QoLS: change from pre- to post-move
Long *et al*^69^ Low secure Female only	Cohort study (4) Follow-up: 3 months	10	Milieu Satisfaction	EssenCES change from pre to post move In-patient satisfaction questionnaire: change from pre to post move
Marks^36^ [unpublished thesis] Medium secure	Retrospective cohort study (4) Follow-up: 4 years	28	Quality of life	QoLS scores during treatment
Trout^84^ [unpublished report] High secure	Cohort study (4) Follow-up: up to 2 years	44	Quality of life Satisfaction	PWI scores: change from pre to post move Service specific evaluation questionnaire using a visual Likert scale via interview: change from pre to post move

QoLS, Quality of Life Scale; EssenCES, Essen Climate Evaluation Scale; PWI, Personal Wellbeing Index.

**Table 6:** Summary of studies presenting data on the outcome domain of patient and carer experience (cross-sectional studies)

Study and setting	Design	N	Outcome sub-domain	Measure/indicator
Langdon *et al*^70^ Medium and low secure	Cross-sectional	18	Milieu	CIES read aloud to patients, scores during treatment
Mansell *et al*^61^ Medium and low secure	Cross-sectional 6-month period	1189	Service satisfaction/complaints Involvement	Number of patient generated complaints per unit over 6-month period, recorded via standardised survey Number of patients with an up-to-date and accessible copy of their own care plan and number of visitors for each patient per unit
Steptoe *et al*^71^ Community	Cross-sectional	28	Quality of life	SOS and LEC scores during treatment
Willetts *et al* (2014)^72^ Medium and low secure	Cross-sectional	45	Milieu	EssenCES scores during treatment

CIES, Correctional Institutions Environment Scale; SOS, Significant Others Scale; LEC, Life experience checklist.

**Table 7:** Initial framework of outcome domains and sub-domains

	Number of studies
Effectiveness	
Discharge outcome/direction of care pathway	26
Delayed discharge/current placement appropriateness	6
Length of hospital stay	22
Readmission (i.e. readmitted to the same setting)	4
Clinical symptom severity (clinician rated)	16
Clinical symptom severity/treatment needs: patient rated	6
Treatment response/engagement	5
Treatment need	2
Reoffending (i.e. charges/reconvictions)	18
‘Offending-like’ behaviour (which did not result in charges)	5
Risk assessment measures	12
Incidents (violence/self-harm)	14
Security need	2
Other	3
Total	139
Patient safety	
Restrictive practices (restraint/relocation/ locked areas/intensive observations)	12
Restrictive practices (seclusion/segregation)	9
Medication (i.e. PRN usage/exceeding BNF prescribing limits)	3
Physical health	1
Premature death/suicide	1
Total	26
Patient experience		
Quality of life	4
Therapeutic milieu	3
Patient experience: involvement	1
Patient experience: satisfaction/complaints	3
Total	11

Each study can be included in more than one sub-domain. PRN, *pro re nata*; BNF, *British National Formulary*.

#### Effectiveness

Fifty-three studies were categorised as presenting data that involved at least a single outcome that attempted to measure effectiveness (Tables 1 and 2). Our analysis led to 12 sub-domains within the effectiveness superordinate domain (Table 7). These included sub-domains such as length of stay, discharge outcome, clinical symptoms, treatment responsiveness, reoffending behaviours and risk assessment.

As a sub-domain, length of stay was considered within 22 studies (Tables 1 and 2), and varied between 1 and 9 years across included studies. However, it was recognised that as a measure of outcome, length of stay is problematic because (a) it tended to be reported for only those who had actually been discharged, rather than the entire in-patient population, and (b) it is complicated because some patients move from one hospital to another, and data may not capture their entire length of stay across all hospitals. As another sub-domain, discharge outcome was considered within 16 studies (Tables 1 and 2) and was defined as moving to an increasing or decreasing level of security within or across forensic hospitals, or discharge to a community-based setting. Several of the included studies focused on delayed discharge^54^^,^^61^^,^^64^^,^^65^ and highlighted the difficulties with finding appropriate accommodation that mitigated risk.

Sixteen studies were judged to have included sub-domains that were classified as falling within the clinical symptom sub-domain and these are detailed in Tables 1 and 2. These included measures that made use of clinician or patient ratings of clinical symptomatology. However, only two studies reported change in clinical symptoms over time for a cohort of patients. A variety of tools were used to index change over time within this sub-domain and included such measures as the Brief Symptom Inventory,^73^ Emotional Problem Scales,^74^ Mini Psychiatric Assessment Schedules for Adults with Developmental Disabilities (mini PAS-ADD),^75^ Health of the Nation Outcome Scale (HoNOS)-Secure^26^^,^^76^ and Clinical Global Impressions Scale.^77^ Treatment responsiveness was also coded as a sub-domain, but it was recognised that this is intertwined with the clinical symptom sub-domain; this was included as a separate sub-domain because it focused on whether a patient was likely to be responsive to treatment efforts, rather than the actual response.

Reoffending and risk were classed as separate sub-domains, with 18 and 12 studies considering variables within these sub-domains, respectively (Tables 1 and 2). Most commonly, studies tended to focus on reoffending using data derived from police or Ministry of Justice records. One study followed up reoffending at 1, 2 and 5 years post-discharge,^35^ whereas another set of studies using the same data-set reported on whether there was reoffending behaviour within the 2 years following discharge from hospital.^78^^,^^79^ Another, based in Australia, considered arrest data and ‘any criminal justice involvement’ following discharge.^80^ A series of studies, based in the community, considered whether treatment within the context of a community-based forensic service led to a reduction in offending behaviours.^23^^,^^30^^–^^33^ It is important to note that many people with intellectual disabilities may not be formally dealt with by criminal justice agencies, and as a consequence, ‘formal’ arrest and conviction data may not be a valid index of reoffending. Hence, the category of ‘reoffending-like behaviour’ described in these studies^17^^,^^22^^,^^29^^,^^30^ is one which is important because it is likely to have increased validity.

Risk also emerged as a likely sub-domain which could be used to index the effectiveness of forensic services for people with intellectual disabilities. The vast majority of these studies reported on their use of structured clinical judgement tools, such as the Historical, Clinical, Risk Management-20 (HCR-20),^20^ with several others considering actuarial risk assessment measures, such as the VRAG,^28^^,^^53^^,^^58^^,^^59^ RM-2000 or STATIC-99.^58^^,^^59^^,^^80^ There was only a single study that considered how changes in scores on a risk assessment tool may relate to treatment outcome from forensic services for people with intellectual disabilities.^37^

#### Patient safety

Eleven studies were categorised as presenting data that were considered to index outcome within the patient safety domain. Premature death was considered by one study, which differentiated between suicide and death associated with natural causes, whereas another study incorporated physical health, an important and relevant sub-domain considering the high rates of morbidity amongst forensic populations, including people with intellectual disabilities.

The five sub-domains that emerged following our analysis also included those related to ‘reactive’ or ‘restrictive’ interventions such as the use of physical interventions and seclusion, *pro re nata* (PRN) medication or a change in observations levels. ‘Reactive’ or ‘restrictive’ interventions fall within the safety domain defined by the Department of Health.^6^^,^^81^ We have adopted the same approach here.

However, there is an overlap with the previously discussed effectiveness domain. ‘Reactive’ or ‘restrictive’ interventions can be construed as proxy variables for behaviour, and their use may correlate with increasing behaviour difficulties. However, they are not an intervention and instead are reactive strategies taken to try to manage behaviour difficulties in the short term to ensure safety. However, medical, psychological and social care interventions developed using a formulation that aim to rehabilitate and/or habilitate are not ‘reactive’, and as such, these would fall within the effectiveness domain. This includes psychological and social interventions, as well as medication prescribed to treat a diagnosed mental illness or distressing symptoms.

#### Patient experience

Within this superordinate domain, 11 studies were categorised as capturing outcomes related to patient experience which were categorised into 4 sub-domains. These were quality of life, therapeutic milieu, patient involvement and patient satisfaction. Four studies included in the review measured quality of life using a number of ratings scales, such as the Quality of Life Questionnaire^36^ or Life Experience Checklist,^71^ whereas three other studies focused on therapeutic milieu or ward atmosphere using either the Correctional Institutions Environment Scale^70^ or the EssenCES Climate Evaluation Scale.^69^^,^^72^ Three studies focused on patient satisfaction in response to service development, and only a single study considered patient involvement as an indicator of outcome.

### Consultation exercise

Following the completion of our systematic review, and the development of our initial outcome framework (Table 7), this was presented to our consultation groups with patients and carers. Revisions were made and the revised outcome framework was used within our Delphi exercise with experts. As with the systematic review, we made use of the three superordinate domains (a) effectiveness, (b) patient safety and (c) patient and carer experience as a framework for our analysis of the data generated from our consultation exercises.

#### Consultation groups

##### Effectiveness

Several patients expressed the view that length of stay should be an important index of outcome; several said they were frustrated because they thought that length of stay was excessive for many patients. However, some carers expressed an alternative view, stating that a shorter length of stay may be problematic and lead to premature discharge. Patients from high security settings were of the opinion that discharge to medium security was indicative of positive progress, whereas for those in medium and low security, discharge to a community-based service was seen as positive. Several carers further discussed how frequent moves between hospitals and wards can be particularly destabilising and may actually be associated with a negative outcome.

The appropriateness of a placement with respect to meeting treatment needs was discussed and considered important by many carers and patients. One stated, ‘I would much rather be further away for eight to nine months [and get the right treatment] than be nearer for 18 months’. Another commented, ‘it is very important for people to go to a place where they are happy, not just because it is closer to family’.

Many commented further about the importance of much needed clinical interventions being available within each service, focusing specifically on psychological treatments and appropriate levels of meaningful activity. Carers spoke about wanting and needing individually tailored care pathways focusing on patient need rather than rigidly designed care pathways that were not based upon a formulation of treatment needs. One said, ‘it has got to be individually led’, and another commented, ‘he needed an individualised package of support which was right for him’. Alongside this, carers also expressed the view that a similar individualised package of support needed to be made available to patients when discharged into the community, with one stating, ‘I worry about the fact that the service wasn’t there in the community…there is so little support in the community’.

Improvements in clinical symptoms and behaviour were recognised by both patients and carers as indicative of positive change. This included quantifiable changes in the frequency of incidents, including improvements in communication and a reduction in angry feelings. Different patients stated, ‘before I wouldn’t engage in conversation and now I’ve learnt different strategies so I don’t kick off so often’, and ‘a reduction in incidents, reduction in restraints, using diversion more frequently, pre-empting incidents’. Carers broadened this by commenting that some patients may not fully understand what they need to achieve to move forward, as there is often too much focus on measuring incidents within services. Some patients also shared this view and stated, ‘there is too much focus on incidents and not on understanding them … taking back to the beginning of the process as opposed to just dealing with what the consequences are’. Carers considered that a family member may be able to make a more nuanced judgement about changes to clinical symptoms because of their long-standing knowledge of the patient. Both patients and carers commented on the importance of engagement with services and therapies as positive indicators of progress within this sub-domain.

Both patients and carers agreed that ‘staying safe’ once discharged was a positive outcome, recognising that a reduction in risk was associated with a positive outcome, and several carers adopted the position of both carer and potential victim, expressing concern that their own safety could be compromised. One carer said, ‘if they said take him home I would be too scared’.

Finally, within the effectiveness superordinate domain, and as an addition to our initial framework, adaptive functioning was considered by patients to be an important indicator of outcome. They talked positively about how they hoped that staying in hospital would bring about improvements in adaptive functioning, such as budgeting, occupational skills and broader life skills. One said, ‘I have been given skills like cooking and cleaning…’. However, several commented that staying in hospital may be associated with a loss of adaptive functioning, and several said they thought they have lost skills. For example, one said, ‘other hospitals let patients get real jobs. I want this to happen in this hospital’, while another commented, ‘since we’ve been locked up here we don’t get a chance to do that sort of thing [budgeting] so you don’t know what to do when you get your money’. One of our carer participants commented, ‘he used to be able to do things. He’s lost those skills since he’s been here’.

##### Patient safety

Patients spoke about how a reduction in aggression and the use of seclusion was a relevant outcome measure, as did a number of carers, whereas patients also spoke about being victimised by other patients in hospital. Some carers further considered that taking medication regularly was an indicator of positive outcome, and they also spoke about how a planned reduction in medication could also be a positive outcome. For example, one commented, ‘if he could come off olanzapine, that would be progress’, whereas another stated, ‘a reduction in PRN medication and other medication is a goal’.

Several carers and patients expressed concern about polypharmacy and side-effects, indicating that they felt this was a restrictive practice and alluding to the possibility that medication may be used to sedate in order to control behaviour; one commented, ‘he’s never been on this amount of medication … he’s so heavily dosed up … if he’s been medicated to manage his behaviour, he’s not learned how to manage his behaviour’. One of the patients strengthened this view by commenting, ‘can you be careful about medication and patients being overdosed’.

##### Patient and carer experience

This superordinate domain was modified as a consequence of our consultation groups in order to incorporate carer experience, alongside the experiences of service users. Carers spoke about whether they were satisfied with the level of care being afforded by their family member and indicated that this was an important measure of outcome. Several spoke about being satisfied with the care being offered by the hospital. One stated, ‘it is a dream come true; the place where he is now, it’s lovely…it’s a dream for places like that to be about’, whereas another commented, ‘the hospital are [sic] fantastic; the staff are fantastic and at long last somebody is realising the amount of problems he has got and that is one of the problems I had before’. Others considered the importance of having a sense of security as a consequence of the quality and responsiveness of care being given to their relative; one stated, ‘not having to be worried about him; if we died tomorrow, services would be there for him and do what was best for him without thinking of the cost’. However, several carers spoke about having to fight or battle for service provision and felt that sometimes services did not listen or involve them appropriately in the care pathway. This was illustrated by the following, ‘there was nothing we could say which would be taken on board … it was very much ‘no’ this is what we think’, and ‘I was asking for help in the community for years before my son was admitted to hospital’.

Patients and carers considered the importance of quality of life as an indicator of positive outcome, and many spoke about having hopes for a job, relationships and involvement in their local communities, with well-integrated high-quality support. Several carers emphasised the importance of high-quality accommodation once a patient was discharged, and one commented, ‘he would be in accommodation that was specifically designed for people with autism, but he had sufficient support with people who actually understood his condition and were able to spot the warning signs so I didn’t have to keep flagging them up’. Another stated, ‘he needs an individual planned package with sufficient staff and appropriate training’. Carers also commented that leaving hospital was not the end of patients’ journeys and spoke about the importance of continuing to monitor outcome and progress over the longer term, rather than view the ‘case as closed’. Others spoke about valuing having in-patient services which could be used in times of crisis; this was illustrated by the following comment, ‘…for him to go back to a secure unit because he’s a danger when he does deteriorate’.

The availability of and engagement with meaningful activity was seen as a potential indicator of positive outcome by both patients and carers. They spoke about having employment, and how increasing engagement in activities could be indicative of improvement. Carers spoke further about the importance of having meaningful activities available within hospital settings and went on to further consider how developing and maintaining social networks are further evidence of a positive outcome. This included developing and maintaining positive relationships with family, friends, and pets, and further included romantic relationships.

##### Changes to our initial outcome framework

A variety of changes to our initial outcome framework were made following the analysis of the data from our consultation groups. This included the incorporation of additional sub-domains or the modification of sub-domains. Specifically, we changed the label of the superordinate domain ‘patient experience’ to ‘patient and carer experience’. Considering the effectiveness superordinate domain, we made changes as follows: (a) treatment response and recovery and clinical symptom severity were modified to include carer ratings of clinical improvement, (b) acquiring adaptive skills was added as a new sub-domain, as was (c) engagement with therapies and services. Within the patient safety superordinate domain, we incorporated (a) safeguarding and victimisation, as a new sub-domain, whereas (b) overuse of medication was strengthened by making reference to unacceptable side-effects and patient satisfaction with prescribed medication. Finally, within the patient and carer experience superordinate domain, we incorporated sub-domains focusing on (a) the carer experience incorporating both communication and involvement, (b) closeness to home area and (c) the level of support and involvement within the community, as well as access to occupational activities. We also included that quality of life could be indexed by either clinicians or the patient.

#### Delphi exercise

Following our revisions to the outcome framework, we completed a two-round Delphi exercise with experts in order to create consensus about the most important outcomes for forensic services for people with intellectual disabilities. None of the sub-domains were rated as ‘not important’ or ‘slightly important’ by the participants. Five sub-domains did not reach consensus at the end of round one, and these were (a) length of stay, (b) security needs, (c) adaptive functioning, (d) clinician-rated quality of life and (e) closeness to home area. Participants were asked to rate five outcomes that they thought were the most important, and length of stay was included within these top five and was therefore retained and taken through to round two.

Six sub-domains received the highest average ratings by experts at the end of round two where a clear consensus emerged. These were (a) discharge outcome, (b) treatment response/engagement, (c) premature death and suicide, (d) therapeutic milieu, (e) meaningful activity and (f) reoffending/offending-like behaviour. However, when asked to indicate their top five sub-domains, participants chose sub-domains exclusively within the effectiveness superordinate domain, and these were (a) clinical symptom severity/treatment needs, (b) reoffending/offending-like behaviour, (c) treatment response/engagement/insight, (d) risk assessment measures and (e) recovery measures/direction of care pathway. Perhaps this is not surprising, considering that all of the participants had current or past clinical responsibility for patients within services.

Integrating the findings, the final most important sub-domains were (a) discharge outcome, (b) recovery measures/direction of care pathway, (c) treatment response/engagement/insight, (d) clinical symptom severity, (e) reoffending/offending-like behaviour, (f) risk assessment, (g) premature death and suicide, (h) therapeutic milieu and (i) access to work and meaningful activity. The findings from the Delphi exercise were considered and synthesised into our findings from the consensus exercises and our systematic review. This led to the emergence of a final outcome framework, and we have identified which aspect of the current project led to the generation of each sub-domain in Table 8. During this process, sub-domains were not removed, but additional sub-domains were added or combined into existing sub-domains that had emerged from our analysis.

**Table 8:** Final framework of outcome domains and sub-domains

	Source
Effectiveness	
Discharge outcome/direction of care pathway	1
Delayed discharge/current placement appropriateness	1
Readmission (i.e. readmitted to hospital or prison)	1
Length of hospital stay	1
Adaptive functioning	1
Clinical symptom severity/treatment needs: patient rated	1
Clinical symptom severity/treatment needs: clinician rated	1
Recovery/engagement/progress on treatment goals: clinician rated	1
Recovery/engagement/progress on treatment goals: patient/carer rated	2
Reoffending (i.e. charges/convictions) on discharge	1
Offending-like behaviour (no CJS involvement) on discharge	1
Incidents (violence/self-harm) (in care setting)	1
Risk assessment measures	1
Security need (i.e. physical/procedural/escort/leave)	1
Patient safety	
Premature death/suicide	1
Physical health	1
Medication (i.e. PRN usage/exceeding BNF limits/side-effects patient rating)	1/2
Restrictive practices (restraint)	1
Restrictive practices (seclusion/segregation)	1
Victimisation/safeguarding	2
Patient and carer experience	
Patient experience: involvement in care	2
Patient experience: satisfaction/complaints	1
Quality of life: patient rated	1
Therapeutic climate	1
Access to work/meaningful activity (where appropriate)	2
Level of support/involvement in community (post discharge)	2
Carer experience: communication with services/involvement in care	2

*Source of domain* Stage 1, systematic review; Stage 2, patient/carer involvement groups, Stage 3 = Delphi; CJS, criminal justice system; PRN, *pro re nata*; BNF, *British National Formulary*.

## Discussion

The aim of this project was to identify the domains that should be used to measure outcome for people with intellectual disabilities and forensic needs. This is a topic relevant to all psychiatrists, particularly following the abuse scandal at a specialist intellectual disability hospital in England, Winterbourne View, and the resulting agenda to care for people with intellectual disabilities within ‘mainstream’ psychiatric services.^11^ Similar issues are of concern around the world as many work toward the social inclusion of people with intellectual disabilities within mainstream services within the health and social care sectors. Our aim was achieved by undertaking a systematic review coupled with a consultation exercise involving patients, carers and experts. The findings revealed a series of important sub-domains spread across three superordinate domains indicative of quality.^12^ These captured a range of clinical and patient safety variables, along with factors measuring both the patient and carer experience of care.

The largest outcome domain was effectiveness, which is not surprising. The sub-domains included were those that captured aspects of the care pathway, along with a focus on clinical symptoms, recovery and a reduction in reoffending. Related variables, such as length of stay, discharge and need for security, were included, but these may not always directly correlate with clinical need. For example, it would be possible for someone who has received successful treatment to remain in hospital due to delayed discharge because of difficulties with the provision of community-based services to manage risk. Further, length of stay in this context, as an indicator of outcome, should be neither too short nor too long, and instead should be ‘just right’ as it should be appropriate to meet the needs of individual patients, adding substantial complexity, especially when considered as a sole indicator of outcome. As such, focusing on multiple sub-domains allows for a richer and more thorough picture of the circumstances surrounding the care being offered to patients within forensic services.

However, consideration as to what ‘effective treatment’ in this context actually looks like requires further exploration, both on an individual patient level and on a wider service level. Only one study^5^ described the nature of the treatment programme that is delivered within the service. Effective treatment is likely to form a combination of appropriate medical, psychological and social intervention, informed by individual clinical formulations, but the availability is likely to vary across services, depending on patient needs. At present, in deciding whether a service is effective, regulatory and commissioning bodies rely on easily measurable process variables (e.g. the existence or otherwise of various policies, and the availability or otherwise of various treatments) rather than paying attention to the more important question of whether any of this is making a difference to the outcome. This is clearly unsatisfactory. Likewise, there are no studies which have looked at the economic evaluation of treatments, a rather surprising finding considering the abundance of anecdote and opinion in this field about costs.^5^ Considering the future, the structure and form of ‘effective treatment’ within forensic services should be clarified and drawn from a robust evidence base, bearing in mind that there are very few clinical trials to identify the most effective intervention across the range of those that are available. As such, greater investment in research investigating the clinical effectiveness of forensic services for people with intellectual disabilities is needed.

There are other sub-domains clustered around safety and the patient and carer experience which we incorporated into our final framework. These are important indicators of the quality of forensic services, but may not always directly relate to clinical effectiveness. Nevertheless, helping to ensure that patients with intellectual disabilities detained in forensic services have a good quality of life, including access to meaningful activity, is a core business of such services. Measuring sub-domains with the broader safety and patient and carer experience domain is clearly important, for all stakeholders, especially patients and their carers. The measurement strategy for these domains could be standardised nationally, bearing in mind that they may not correlate directly with clinical effectiveness.

Related to this, the addition of a series of consultation exercises, alongside our systematic review, adds a particular strength to our project. This helped to ensure that we adequately captured the views of all stakeholders and appropriately synthesised them into our final framework. It is important to note that although experts tended to focus on clinical outcomes, patients and carers tended to focus more upon the quality of service provision, and the experience of receiving a service, alongside clinical outcomes. As such, it became important to ensure that these findings formed part of our final outcome framework.

Contrasting our framework with that developed by Fitzpatrick *et al*,^8^ there are both similarities and differences. Fitzpatrick *et al*^8^ grouped outcome measures across a variety of similar domains, such as recidivism, service outcomes, mental state, compliance, satisfaction and substance misuse, among others. They were able to successfully review a variety of specific outcome measures that would enable measurement across these domains, whereas in our study there are relatively fewer instruments that have been standardised for use with people with intellectual disabilities across the sub-domains we have included within our framework. At the same time, there were some noted differences between our framework and that reported by Fitzpatrick *et al*.^8^ For example, substance misuse did not feature explicitly in our framework, but nevertheless is an issue for many with intellectual disabilities, and would fall easily within our Incidents sub-domain. Conversely, there were specific sub-domains that we included which did not appear within the framework reported by Fitzpatrick *et al*,^8^ such as adaptive functioning, access to meaningful activity, as well as the use of restrictive practices, which no doubt are all issues for those with forensic mental health problems and are likely to be more salient with services for people with intellectual disabilities.

### Clinical implications

The findings from the current project have direct relevance to recent government initiatives, including Building the Right Support^7^ and the new National Service Model^83^ that were developed and published in response to the institutional abuse that took place at Winterbourne View in England.^82^ For many years, there has been a focus on ensuring that people with intellectual disabilities are afforded good quality care within their own communities, rather than in hospital, and the abuse that occurred at Winterbourne View has reignited the drive to ensure that people with intellectual disabilities are not unnecessarily kept in hospital and other restrictive environments, recognising at the same time that some people with intellectual disabilities do need appropriate hospital care from time to time, depending upon their needs. The new National Service Model incorporated hospital admission, which should be integrated within community-based teams, alongside active, clear and robust discharge planning. In order to achieve these aims, services need to be able to measure outcomes, and for those who are admitted to in-patient forensic services, including forensic rehabilitation services, our framework of outcomes should be used by hospitals to index change, as well as service quality. Further, our work has the potential to strengthen current initiatives, such as the Quality Network for Forensic Services (http://www.rcpsych.ac.uk/quality/quality,accreditationaudit/forensicmentalhealth/templatehomepage.aspx), when used within forensic services for people with intellectual disabilities where there is a focus on ensuring practice standards are agreed and met.

Care and treatment reviews, a further initiative created by NHS England following Winterbourne View, involve reviewing the care within a hospital in order to make a judgement about whether an individual is receiving the right care within the right environment. Each review involves a service commissioner and at least two expert advisors, one being a carer or patient. For patients who are within in-patient forensic services, it would be valuable for care and treatment reviews to be structured around our outcome framework. This would help ensure that decisions about care are based on the research evidence and on indicators that are considered to measure change appropriately, helping to ensure the process is robust. One of the further important findings from our work is that we have integrated the findings from the evidence base which was used as the springboard to develop our framework. Although there are difficulties with many of the included studies, what was apparent was the absence of a focus on recovery and exploration of the subjective meaning of recovery in this context. Alongside this, many of the studies were small and very few longitudinal studies drawing on a well-developed outcomes framework have been completed, which is both clearly and sorely needed.

### Limitations

All of these recommendations need to be balanced against several weaknesses associated with this study. First, our findings from the systematic review are based on the research evidence. Inherently, our findings from the systematic review are only as robust as the quality of the research that was reviewed. The predominant issue with many of the studies that were included was that few were longitudinal studies measuring outcomes, demonstrating that these outcomes had validity and reliability as an outcome indicator. Related to this, because of the marked variation across studies in terms of methodology, it became impossible to find a suitably reliable and valid tool that would index quality in this context. Moreover, if we had been able to measure study quality, this would not have altered the weight put on one study as opposed to another, because the focus was on the domains and how those domains were measured.

Although this is problematic, it is attenuated by the consultation exercises with patients, carers and experts. The patients included within our focus groups are vulnerable, detained under the Mental Health Act, and are often not given a voice. They directly contributed to the development of our outcomes, telling us what was important to them, as users of the services. Our findings from the consultation exercises were incorporated into our methods which helped to ensure that our findings were shaped carefully by those affected by our findings, which in turn increased validity. The second weakness is that the findings from our consensus exercises are based upon the views of a group of individuals, and only four carers were included; our findings may have been enhanced with a larger number of carers, but the content had become repetitive suggesting we had reached content saturation. Although we attempted to capture the views of a variety of patients, carers and experts, it is certainly possible that had we asked a different group of patients, carers and experts, different issues may have emerged from our analysis. Third, as our study included participants from the UK, there is a question as to whether the findings are generalisable to healthcare systems in other countries. However, we would anticipate that the findings have implications within other countries offering similar services and could be used to inform further research within similar hospitals and services in other parts of the world.

Finally, and looking forward to the future, further work is needed to investigate the reliability and validity of our outcomes framework. This may lead to a reduction in the number of sub-domains found within our current outcomes framework, which would increase the probability that services would integrate the framework into their services. Related to this, it is important to consider that individual-level outcomes are likely to be very important when indexing recovery, and further work is needed as to how these are measured across services, because they are likely to be associated with local clinical practices which may be idiographic and vary from service to service. However, a degree of standardisation would be valuable when monitoring and improving service outcomes, and specifying the method of measurement across our sub-domains is an important next step. Together, our framework should have a beneficial impact on improving both service quality and patient outcomes, whereas it would also allow for the creation of a national minimum data-set, specific to these services, which could be used to track patient outcomes and help develop and refine care pathways. Considering future research, it is now appropriate to consider the likely instruments that could be used to measure outcomes, allowing us to trial this framework within existing hospital care pathways.

## References

[1] Health and Social Care Information Centre. *Learning Disabilities Census Report*. HSCIC, 2013.

[2] Devapriam J, Rosenbach A, Alexander R. In-patient services for people with intellectual disability and mental health or behavioural difficulties. *BJPscyh Adv* 2015; 21: 116–23.

[3] Royal College of Psychiatrists’ Faculty of Psychiatry of Intellectual Disability. *People with Learning Disability and Mental Health, Behavioural or Forensic Problems: The Role of In-Patient Services*. RCPsych, 2013.

[4] Esan F, Chester V, Gunaratna IJ, Hoare S, Alexander RT. The clinical, forensic and treatment outcome factors of patients with autism spectrum disorder treated in a forensic intellectual disability service. *J Appl Res Intellect Disabil* 2015; 28: 193–200. 25379816 10.1111/jar.12121

[5] Alexander R, Hiremath A, Chester V, Green F, Gunaratna I, Hoare S. Evaluation of treatment outcomes from a medium secure unit for people with intellectual disability. *Adv Ment Health Intellect Disabil* 2011; 5: 22–32.

[6] Department of Health. *The NHS Outcomes Framework 2015/16*. Department of Health, 2014.

[7] NHS England. *Building the Right Support: A National Plan to Develop Community Services and Close Inpatient Facilities for People with a Learning Disability and/or Autism who Display Behaviour that Challenges, Including those with a Mental Health Condition*. NHS England, 2015.

[8] Fitzpatrick R, Chambers J, Burns T, Doll H, Fazel S, Jenkinson C, et al A systematic review of outcome measures used in forensic mental health research with consensus panel opinion. *Health Technol Assess* (*Rockv*) 2010; 14: 1–94. 10.3310/hta14180 20350473

[9] Gilbody SM, House AO, Sheldon TA. *Outcomes Measurement in Psychiatry: A Critical Review of Outcomes Measurement in Psychiatric Research and Practice*. Centre for Reviews and Dissemination, 2003.

[10] Leamy M, Bird V, Le Boutillier C, Williams J, Slade M. Conceptual framework for personal recovery in mental health: systematic review and narrative synthesis. *Br J Psychiatry* 2011; 199: 445–2. 22130746 10.1192/bjp.bp.110.083733

[11] Department of Health. *Transparency in Outcomes: A Framework for the NHS*. Department of Health, 2010.

[12] Hassiotis A, Hall I. Behavioural and cognitive-behavioural interventions for outwardly-directed aggressive behaviour in people with learning disabilities. *Cochrane Database Syst Rev* 2004; 4: CD003406. 10.1002/14651858.CD003406.pub2 15495051

[13] Duggan C. The empirical basis of sex offender treatment effectiveness. *Sex Offender Treat* 2014; 9: 1–13.

[14] Hsieh H-F, Shannon SE. Three approaches to qualitative content analysis. *Qual Health Res* 2005; 15: 1277–88. 16204405 10.1177/1049732305276687

[15] Linstone HA, Turoff M. *The Delphi Method : Techniques and Applications*. Addison-Wesley, 1975.

[16] Murphy MK, Black NA, Lamping DL, McKee CM, Sanderson CF, Askham J, et al Consensus development methods, and their use in clinical guideline development. *Health Technol Assess* 1988; 2(3): i–iv, 1–88. 9561895

[17] Alexander RT, Crouch K, Halstead S, Piachaud J, Piachaud J, Singh I, et al Long-term outcome from a medium secure service for people with intellectual disability. *J Intellect Disabil Res* 2006; 50: 305–15. 16507035 10.1111/j.1365-2788.2006.00806.x

[18] Alexander RT, Green FN, O’Mahony B, Gunaratna IJ, Gangadharan SK, Hoare S. Personality disorders in offenders with intellectual disability: a comparison of clinical, forensic and outcome variables and implications for service provision. *J Intellect Disabil Res* 2010; 54: 650–8. 20136682 10.1111/j.1365-2788.2010.01248.x

[19] Alexander RT, Chester V, Green FN, Gunaratna I, Hoare S. Arson or fire setting in offenders with intellectual disability: clinical characteristics, forensic histories, and treatment outcomes. *J Intellect Dev Disabil* 2015; 40: 189–97.

[20] Alexander RT, Chester V, Gray NS, Snowden RJ. Patients with personality disorders and intellectual disability – closer to personality disorders or intellectual disability? A three-way comparison. *J Forens Psychiatry Psychol* 2012; 23: 435–51.

[21] Ayres M, Roy A. Supporting people with complex mental health needs to get a life! The role of the Supported Living Outreach Team. *Tizard Learn Disabil Rev* 2009; 14: 29–39.

[22] Barron P, Hassiotis A, Banes J. Offenders with intellectual disability: a prospective comparative study. *J Intellect Disabil Res* 2004; 48: 69–76. 14675234 10.1111/j.1365-2788.2004.00581.x

[23] Benton C, Roy A. The first three years of a community forensic service for people with a learning disability. *Br J Forensic Pract* 2008; 10: 4–12.

[24] Butwell M, Jamieson E, Leese M, Taylor P. Trends in special (high-security) hospitals. 2: Residency and discharge episodes, 1986–1995. *Br J Psychiatry* 2000; 176: 260–5. 10755074 10.1192/bjp.176.3.260

[25] Day K. A hospital-based treatment programme for male mentally handicapped offenders. *Br J Psychiatry* 1988; 153: 635–44. 2855609 10.1192/bjp.153.5.635

[26] Dickens G, Sugarman P, Picchioni M, Long C. HoNOS-secure: tracking risk and recovery for men in secure care. *Br J Forensic Pract* 2010; 12: 36–46.

[27] Fitzgerald S, Gray NS, Taylor J, Snowden RJ. Risk factors for recidivism in offenders with intellectual disabilities. *Psychol Crime Law* 2011; 17: 43–58.

[28] Gray NS, Fitzgerald S, Taylor J, Macculloch MJ, Snowden RJ. Predicting future reconviction in offenders with intellectual disabilities: the predictive efficacy of VRAG, PCL-SV, and the HCR-20. *Psychol Assess* 2007; 19: 474–9. 18085940 10.1037/1040-3590.19.4.474

[29] Halstead S, Cahill A, Fernando L, Isweran M. Discharges from a learning-disability medium secure unit: what happens to them? *Br J Forensic Pract* 2001; 3: 11–21.

[30] Lindsay WR, Smith AHW, Law J, Quinn K, Anderson A, Smith A, et al A treatment service for sex offenders and abusers with intellectual disability: characteristics of referrals and evaluation. *J Appl Res Intellect Disabil* 2002; 15: 166–74.

[31] Lindsay WR, Smith AHW, Law J, Quinn K, Anderson A, Smith A, et al Sexual and nonsexual offenders with intellectual and learning disabilities: a comparison of characteristics, referral patterns, and outcome. *J Interpers Violence* 2004; 19: 875–90. 15231027 10.1177/0886260504266884

[32] Lindsay WR, Steele L, Smith AHW, Quinn K, Allan R. A community forensic intellectual disability service: twelve year follow up of referrals, analysis of referral patterns and assessment of harm reduction. *Leg Criminol Psychol* 2006; 11: 113–30.

[33] Lindsay WR, Steptoe L, Wallace L, Haut F, Brewster E. An evaluation and 20-year follow-up of a community forensic intellectual disability service. *Crim Behav Ment Health* 2013; 23: 138–49. 23595864 10.1002/cbm.1859

[34] Lindsay WR, Holland T, Wheeler JR, Carson D, O’Brien G, Taylor JL, et al Pathways through services for offenders with intellectual disability: a one- and two-year follow-up study. *Am J Intellect Dev Disabil* 2010; 115: 250–62. 20441394 10.1352/1944-7558-115.3.250

[35] Linhorst DM, McCutchen TA, Bennett L. Recidivism among offenders with developmental disabilities participating in a case management program. *Res Dev Disabil* 2003; 24: 210–30. 12742389 10.1016/s0891-4222(03)00029-5

[36] Marks K. *Assessing Risk and Outcomes in Offenders Detained in Intellectual Disability and Mental Health Medium Secure Units in the United Kingdom*. University of Birmingham, 2011.

[37] Morrissey C, Taylor J. Changes in personality disorder traits following 2 years of treatment in a secure therapeutic community milieu. *J Ment Health Res Intellect Disabil* 2014; 7: 323–36.

[38] Morrissey C, Beeley C, Milton J. Longitudinal HCR-20 scores in a high-secure psychiatric hospital. *Crim Behav Ment Health* 2014; 24: 169–80. 24265120 10.1002/cbm.1893

[39] Morrissey C, Mooney P, Hogue TE, Lindsay WR, Taylor JL. Predictive validity of the PCL-R for offenders with intellectual disability in a high security hospital: treatment progress. *J Intellect Dev Disabil* 2007; 32: 125–33. 17613683 10.1080/13668250701383116

[40] Morrissey C, Hobson B, Faulkner E, James T. Outcomes from the National High Secure Learning Disability Service: findings and challenges. *Adv Ment Health Intellect Disabil* 2015; 9: 116–23.

[41] Palucka AM, Raina P, Liu S, Lunsky Y. The clinical profiles of forensic inpatients with intellectual disabilities in a specialized unit. *J Learn Disabil Offending Behav* 2012; 3: 219–27.

[42] Reed S, Russell A, Xenitidis K, Murphy DGM. People with learning disabilities in a low secure in-patient unit: Comparison of offenders and non-offenders. *Br J Psychiatry* 2004; 185: 499–504. 15572741 10.1192/bjp.185.6.499

[43] Xenitidis KI, Henry J, Russell AJ, Ward A, Murphy DG. An inpatient treatment model for adults with mild intellectual disability and challenging behaviour. *J Intellect Disabil Res* 1999; 43: 128–34. 10221793 10.1046/j.1365-2788.1999.00184.x

[44] Lindsay WR, Smith AHW, Quinn K, Anderson A, Smith A, Allan R, et al Women with intellectual disability who have offended: characteristics and outcome. *J Intellect Disabil Res* 2004; 48: 580–90. 15312059 10.1111/j.1365-2788.2004.00627.x

[45] Lindsay WR, Elliot SF, Astell A. Predictors of sexual offence recidivism in offenders with intellectual disabilities. *J Appl Res Intellect Disabil* 2004; 17: 299–305.

[46] Ajmal MA. Self-esteem and mental health in a forensic learning disabilities setting. *Pakistan J Soc Clin Psychol* 2008; 4: 47–58.

[47] Beer D, Turk V, McGovern P, Gravestock SM, Brooks D, Barnett L, et al Characteristics of patients exhibiting severe challenging behaviour in low secure mental health and mild learning disabilities units. *J Psychiatr Intensive Care* 2005; 1: 29.

[48] Beer D, Spiller MJ, Pickard M, Gravestock S, Mcgovern P, Leese M, et al Low secure units: factors predicting delayed discharge. *J Forens Psychiatry Psychol* 2005; 16: 621–37.

[49] Chaplin E, Eyeoyibo M, Wright S, Xenitidis K, McCarthy J. Historical and clinical items of the HCR-20 as predictors of risk within an intellectual disability population. *Adv Ment Health Intellect Disabil* 2015; 9: 62–9.

[50] Chilvers J, Thomas C. Do male and female forensic patients with learning disabilities differ on subscales of the Novaco Anger Scale and Provocation Inventory (NAS-PI)? *J Learn Disabil Offending Behav* 2011; 2: 84–97.

[51] Crossland S, Burns M, Leach C, Quinn P. Needs assessment in forensic learning disability. *Med Sci Law* 2005; 45: 147–53. 15895641 10.1258/rsmmsl.45.2.147

[52] Dickens G, Picchioni M, Long C. Aggression in specialist secure and forensic inpatient mental health care: incidence across care pathways. *J Forensic Pract* 2013; 15: 206–17.

[53] Fitzgerald S, Gray N, Alexander RT, Bagshaw R, Chesterman P, Huckle P, et al Predicting institutional violence in offenders with intellectual disabilities: the predictive efficacy of the VRAG and the HCR‐20. *J Appl Res Intellect Disabil* 2013; 26: 384–93. 23925961 10.1111/jar.12032

[54] Hall I, Yacoub E, Boast N, Bates R, Stamps R, Holder S, et al Secure inpatient services: a needs assessment. *J Intellect Disabil Offending Behav* 2014; 5: 38–53.

[55] Hogue TE, Mooney P, Morrissey C, Steptoe L, Johnston S, Lindsay WR, et al Emotional and behavioural problems in offenders with intellectual disability: comparative data from three forensic services. *J Intellect Disabil Res* 2007; 51: 778–85. 17803496 10.1111/j.1365-2788.2006.00938.x

[56] Johnson P. The prevalence of low self-esteem in an intellectually disabled forensic population. *J Intellect Disabil Res* 2012; 56: 317–25. 21726326 10.1111/j.1365-2788.2011.01447.x

[57] Kellett S, Beail N, Newman DW, Frankish P. Utility of the brief symptom inventory in the assessment of psychological distress. *J Appl Res Intellect Disabil* 2003; 16: 127–34.

[58] Lindsay WR, Hogue TE, Taylor JL, Steptoe L, Mooney P, O’Brien G, et al Risk assessment in offenders with intellectual disability: a comparison across three levels of security. *Int J Offender Ther Comp Criminol* 2008; 52: 90–111. 18174529 10.1177/0306624X07308111

[59] Lindsay W, Carson D, O’Brien G, Holland AJ, Johnston S, Taylor JL, et al The relationship between assessed risk and service security level for offenders with intellectual disability. *J Forens Psychiatry Psychol* 2010; 21: 537.

[60] Lofthouse RE, Lindsay WR, Totsika V, Hastings RP, Roberts D. Dynamic risk and violence in individuals with an intellectual disability: tool development and initial validation. *J Forens Psychiatry Psychol* 2014; 25: 288–306.

[61] Mansell J, Ritchie F, Dyer R. Health service inpatient units for people with intellectual disabilities and challenging behaviour or mental health problems. *J Appl Res Intellect Disabil* 2010; 23: 552–9.

[62] McMillan D, Hastings RP, Coldwell J. Clinical and actuarial prediction of physical violence in a forensic intellectual disability hospital: a longitudinal study. *J Appl Res Intellect Disabil* 2004; 17: 255–65.

[63] O’Shea LE, Picchioni MM, McCarthy J, Mason FL, Dickens GL. Predictive validity of the HCR-20 for inpatient aggression: the effect of intellectual disability on accuracy. *J Intellect Disabil Res* 2015; 59: 1042–54. 25683589 10.1111/jir.12184

[64] Perera C, Simpson N, Douds F, Campbell M. A survey of learning disability inpatient services in Scotland in 2007. *J Intellect Disabil* 2009; 13: 161–71. 19628535 10.1177/1744629509339091

[65] Thomas SD, Dolan M, Johnston S, Middleton H, Harty MA, Carlisle J, et al Defining the needs of patients with intellectual disabilities in the high security psychiatric hospitals in England. *J Intellect Disabil Res* 2004; 48: 603–10. 15312061 10.1111/j.1365-2788.2004.00629.x

[66] Uppal G, McMurran M. Recorded incidents in a high-secure hospital: a descriptive analysis. *Crim Behav Ment Health* 2009; 19: 265–76. 19780021 10.1002/cbm.741

[67] Mason T. Seclusion and learning disabilities: research and deduction. *Br J Dev Disabil* 1996; 42: 149–59.

[68] Fish R, Lobley J. Evaluating a forensic service for people with learning disabilities: comparing approaches. *J Intellect Disabil* 2001; 5: 97–109.

[69] Long CG, Bell N, Carr A, Cairns L, Webb A, Collins L. The benefits of environmental change in a secure service for people with intellectual disabilities. *Adv Ment Heal Intellect Disabil* 2014; 8: 309–20.

[70] Langdon PE, Swift A, Budd R. Social climate within secure inpatient services for people with intellectual disabilities. *J Intellect Disabil Res* 2006; 50: 828–36. 16999782 10.1111/j.1365-2788.2006.00847.x

[71] Steptoe L, Lindsay WR, Forrest D, Power M. Quality of life and relationships in sex offenders with intellectual disability. *J Intellect Dev Disabil* 2006; 31: 13–9. 16766318 10.1080/13668250500488652

[72] Willets L, Mooney P, Blagden N. Social climate in learning disability services. *J Intellect Disabil Offending Behav* 2014; 5: 24–37.

[73] Leonard RD. *Brief Symptom Inventory* (BSI^®^). Pearson Clinical, 1993.

[74] Prout HT, Strohmer DC. *Emotional Problems Scales. Professional Manual for the Behaviour Rating Scales and Self-Report Inventory*. Psychological Assessment Resources, 1991.

[75] Moss S. *The Mini PAS-ADD Handbook*. Pavilion, 2014.

[76] Dickens G, Sugarman P, Walker L. HoNOS-secure: a reliable outcome measure for users of secure and forensic mental health services. *J Forens Psychiatry Psychol* 2007; 18: 507–14.

[77] Guy W. *ECDEU Assessment Manual for Psychopharmacology*. US Department of Health, Education, and Welfare Public Health Service Alcohol, Drug Abuse, and Mental Health Administration, 1976.

[78] Douglas KS, Hart SD, Webster CD, Belfrage H, Guy LS, Wilson CM. Historical-clinical-risk management-20, version 3 (HCR-20V3): development and overview. *Int J Forensic Ment Health* 2014; 13: 93–108.

[79] Harris GT, Rice, ME, Camilleri JA. Applying a forensic actuarial assessment (the violence risk appraisal guide) to non-forensic patients. *J Interpers Violence* 2004; 19: 1063–74. 15296617 10.1177/0886260504268004

[80] Hanson, RC, Thornton D. Static 99: Improving Actuarial Risk Assessments for Sex Offenders. In *Prediction of Criminal Behaviour*. Public Safety Canada, 1999.

[81] Department of Health. *Positive and Proactive Care: Reducing the Need for Restrictive Interventions*. Department of Health, 2014.

[82] Glover G, Brown I, Hatton C. How psychiatric in-patient care for people with learning disabilities is transforming after Winterbourne View. *Tizard Learn Disabil Rev* 2014; 19: 146–9.

[83] NHS England. *Supporting People with a Learning Disability and/or Autism who Display Behaviour that Challenges, Including those with a Mental Health Condition. Service Model for Commissioners of Health and Social Care Services*. NHS England, 2015.

[84] Trout S. National High Secure Learning Disability Service, Rampton Hospital: Service Evaluation. Patient Focus, 2011 [unpublished report].

